# Conservative Management of Odontogenic Fibromyxoma of the Maxilla: A Case Report

**DOI:** 10.7759/cureus.59763

**Published:** 2024-05-06

**Authors:** Aditya Hurkat, Gidean A Sundaram, Vinod K Krishna, Murugesan Krishnan, Santhosh P Kumar

**Affiliations:** 1 Oral and Maxillofacial Surgery, Saveetha Dental College and Hospitals, Saveetha Institute of Medical and Technical Sciences, Saveetha University, Chennai, IND

**Keywords:** conservative management, maxilla, myxofibroma, odotogenic myxoma, odontogenic tumour, odontogenic fibromyxoma

## Abstract

Odontogenic fibromyxoma typically presents as painless swelling in the jaw, and clinically, it grows slowly, becoming benign and asymptomatic. It causes the cortical plates to expand gradually, which leads to mobility and drifting of the teeth. Root resorption is also common. The tumor is locally aggressive in nature. It is also known to have a high recurrence rate. We present the case of a 30-year-old female patient who was diagnosed and treated for odontogenic fibromyxoma of the maxilla conservatively with enucleation. The radiograph showed a multilocular lesion, which can be confused with ameloblastoma, aneurysmal bone cyst, or odontogenic keratocyst. Hence, with proper clinical, radiographic, and histopathological examination, a correct diagnosis can be made and adequate treatment can be planned.

## Introduction

Benign odontogenic tumors of the jaw are known to originate from the odontogenic epithelium and ectomesenchyme cells. Odontogenic fibromyxoma is a benign, unencapsulated, locally invasive tumor with dense fibrous connective tissue [1,2]. Virchow, in the year 1863, first used the term myxoma. Then later, the term was replaced by fibromyxoma, given by Dietrich et al. [3]. In 2005, WHO categorized odontogenic myxomas as ectomesenchymal tumors, either including or excluding the odontogenic epithelium [4]. WHO did, however, classify odontogenic myxoma as central and peripheral forms of mesenchymal odontogenic tumors later in 2017. Comparatively, peripheral odontogenic myxomas are less aggressive, and they are usually encapsulated, unlike central myxomas, which are typically nonencapsulated tumors that have the ability to infiltrate into nearby bone [5].

Heart, skin, subcutaneous tissue, and bone can all have myxomas; however, jaw myxomas are uncommon, constituting only about 1-3% of all jaw tumors and cysts. Frequently seen in the mandible rather than the maxilla, during the third and fifth decades of life, they are more prevalent in females [6]. Radiographically, they can be visualized as small unilocular to large multilocular radiolucent lesions with a well-defined border. Most of them are multilocular, showing the characteristic “tennis racquet” appearance [7]. Clinically, they present as slow-growing lesions with the ability to infiltrate local bone and soft tissues, leading to cortical bone expansion and root resorption, causing tooth movement [8]. On histological examination, odontogenic myxoma consists of spindle-shaped cells embedded in an intercellular matrix that is primarily made of mucoid. It shows no encapsulation, with scattered residual bony trabeculae [9]. Lesion size can vary, and therapy is tailored accordingly. Smaller myxomas are treated conservatively with curettage and enucleation, whereas larger tumors may require extensive excision due to a 25% recurrence rate [10].

## Case presentation

**Figure 1:**
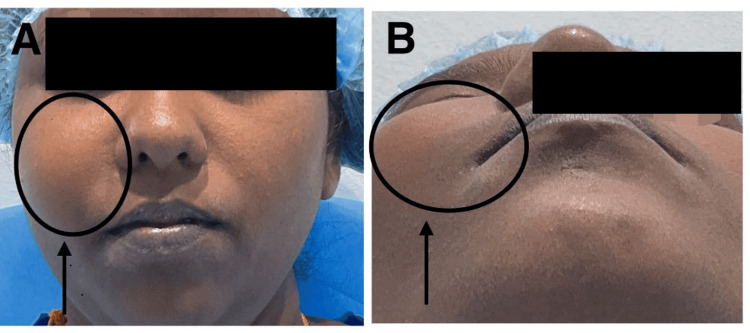
Preoperative photographs (A) Frontal view. (B) Worm’s-eye view.

**Figure 2:**
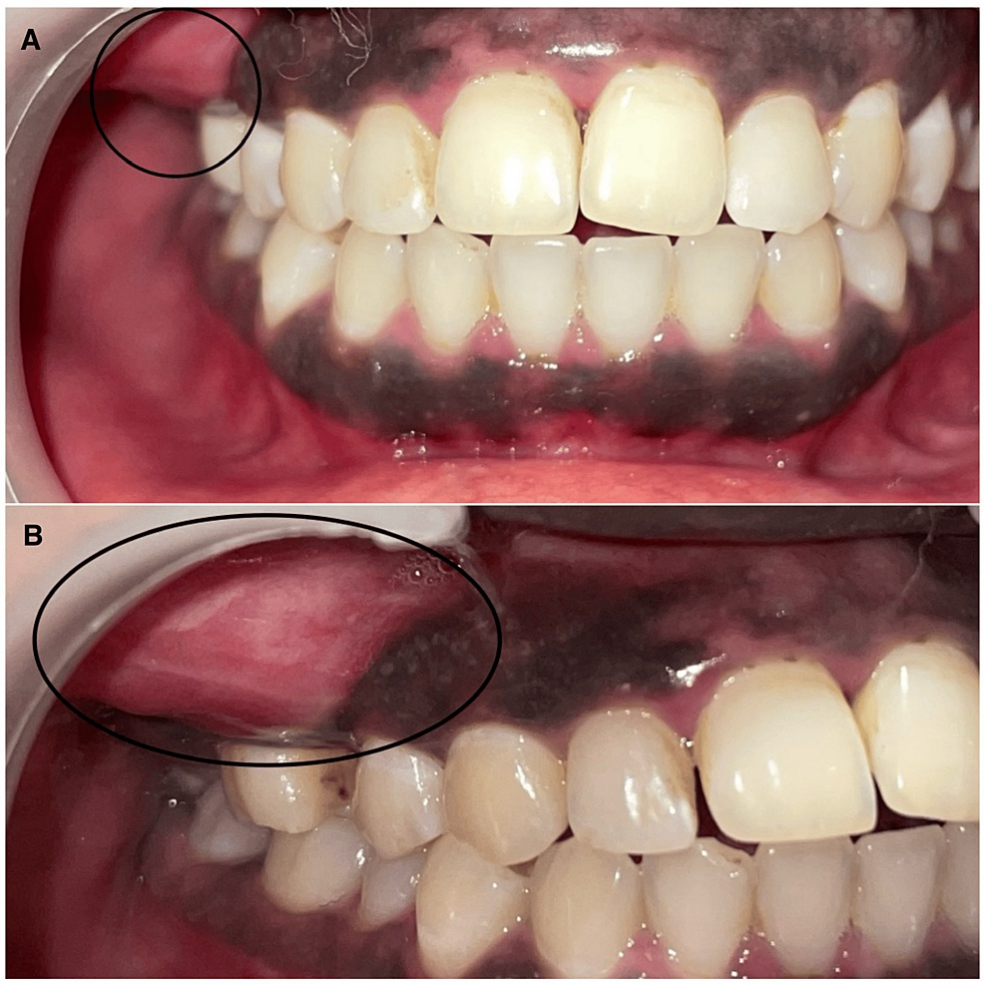
Preoperative intraoral photographs (A) Frontal view. (B) Right-side view.

A 30-year-old female patient presented to the Department of Oral and Maxillofacial Surgery with a chief complaint of pain and swelling in the upper right cheek region and tooth for three years. The swelling had gradually increased in size over the course of three years. Presently, she complained of pain in the upper right back tooth region for the last seven days and mentioned a history of extraction in the same region six months ago. No similar swelling was noted elsewhere in the body. There were no other significant medical, surgical, or dental histories. There was no familial history of similar swelling. Clinical examination revealed extraoral swelling on the right side of the face, from the infraorbital region to the corner of the mouth, extending 4 cm from the tragus, and measuring 4 cm by 6 cm in size (Figures 1, 2). Intraoral swelling was noted in relation to the upper right buccal sulcus. No pus discharge or ulceration was observed. On palpation, the swelling was bony and hard in consistency, nontender, and nonfluctuating.

**Figure 3:**
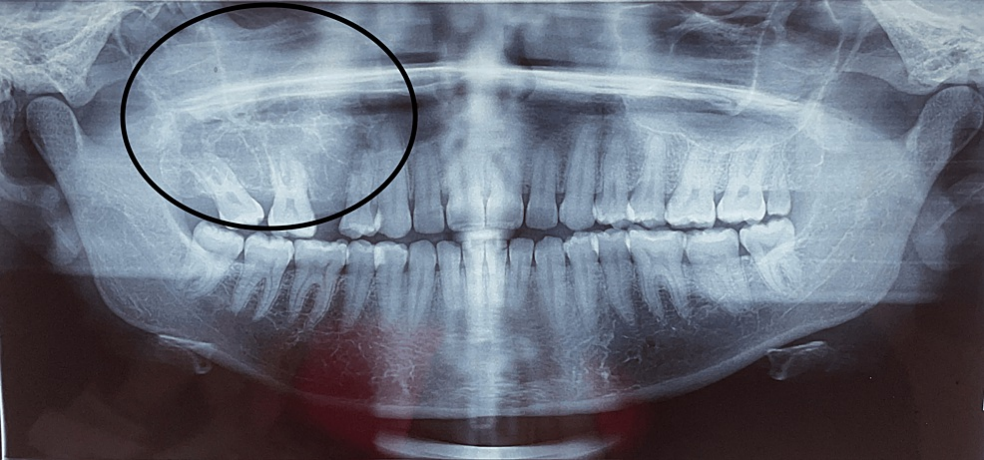
Preoperative orthopantomogram depicting “tennis racquet-like” appearance

**Figure 4:**
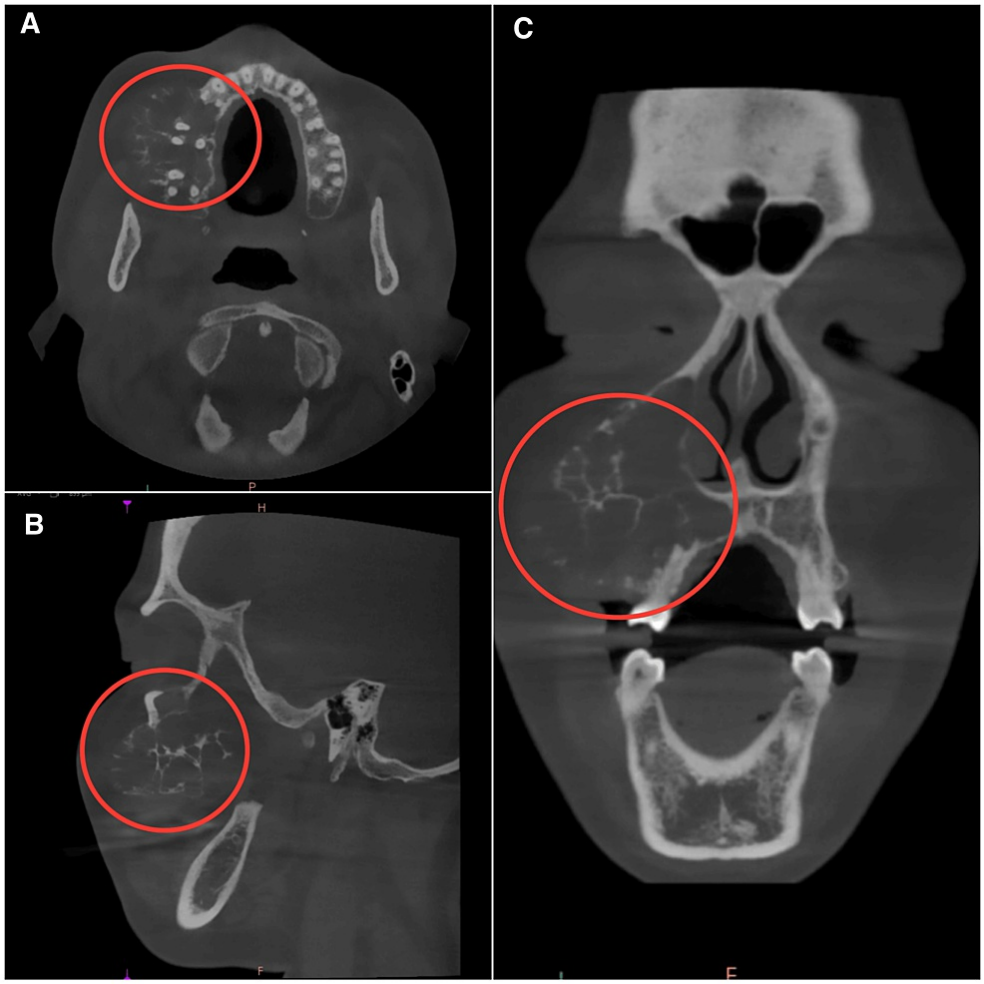
Preoperative CBCT showing bony expansion extending to the infraorbital rim (A) Axial view. (B) Coronal view. (C) Sagittal view.

The radiograph revealed osteolytic areas involving the right maxillary alveolus, sinus, palate, orbit, expansion, and destruction of buccal and palatal cortices, thin radiopaque septae, and displacement and root resorption of teeth 14 and 16 (Figures 3, 4).

Needle aspiration yielded negative results. An incisional biopsy was performed, and the reports revealed multiple H&E-stained sections with loose myxoid connective tissue stroma admixed with collagen fibers, suggesting odontogenic fibromyxoma of the maxilla.

**Figure 5:**
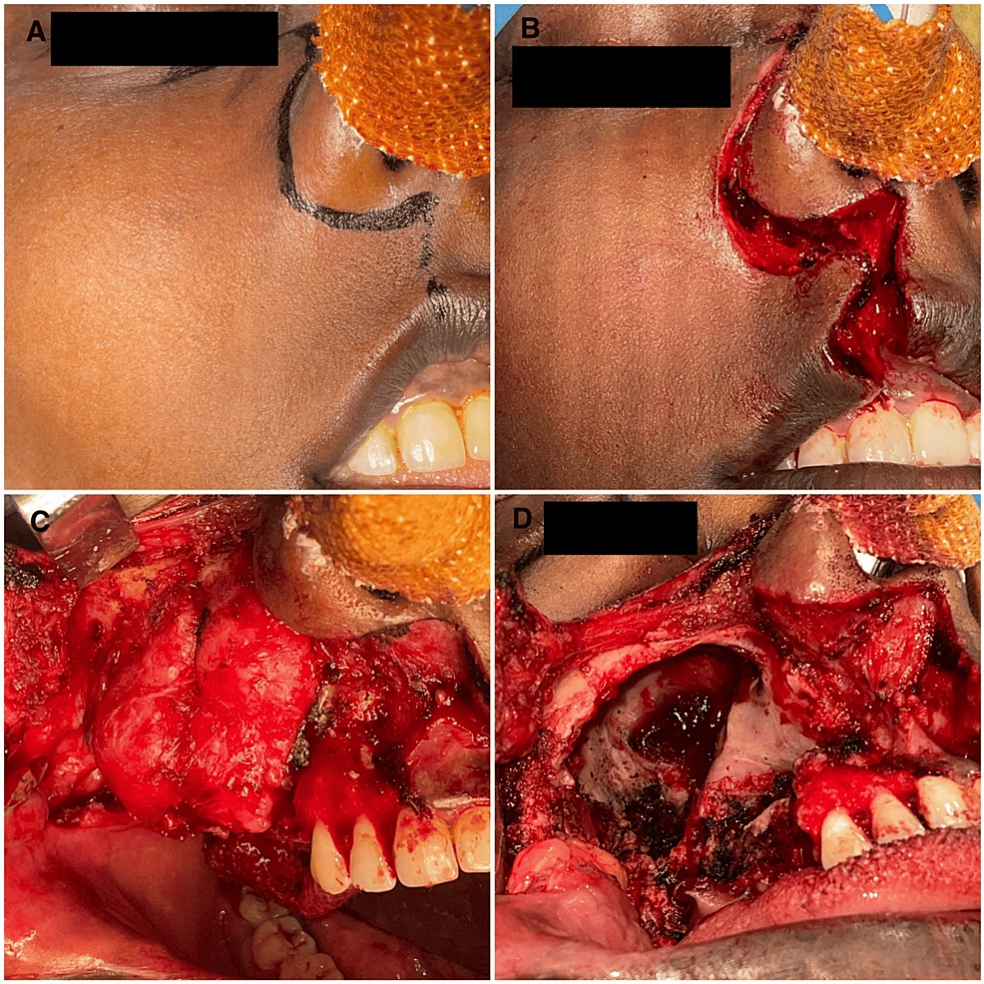
Intraoperative photos (A) Incision marking. (B) Incision. (C) Mucoperiosteal flap elevation and exposure. (D) Surgical defect postexcision.

**Figure 6:**
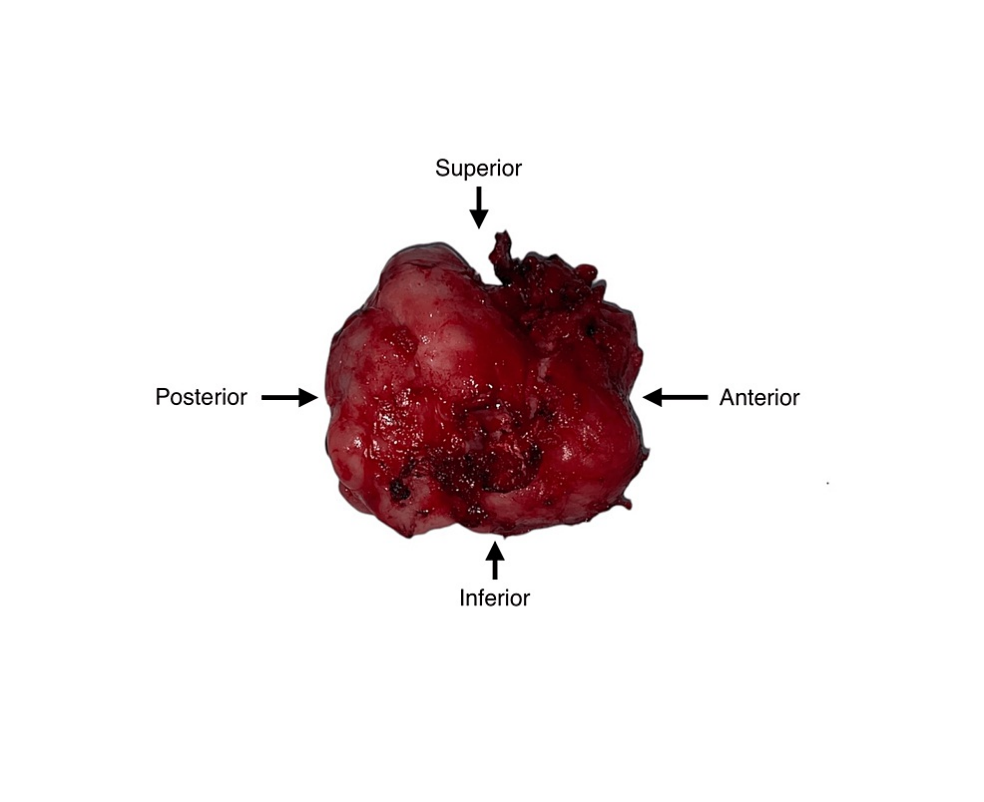
Specimen

**Figure 7:**
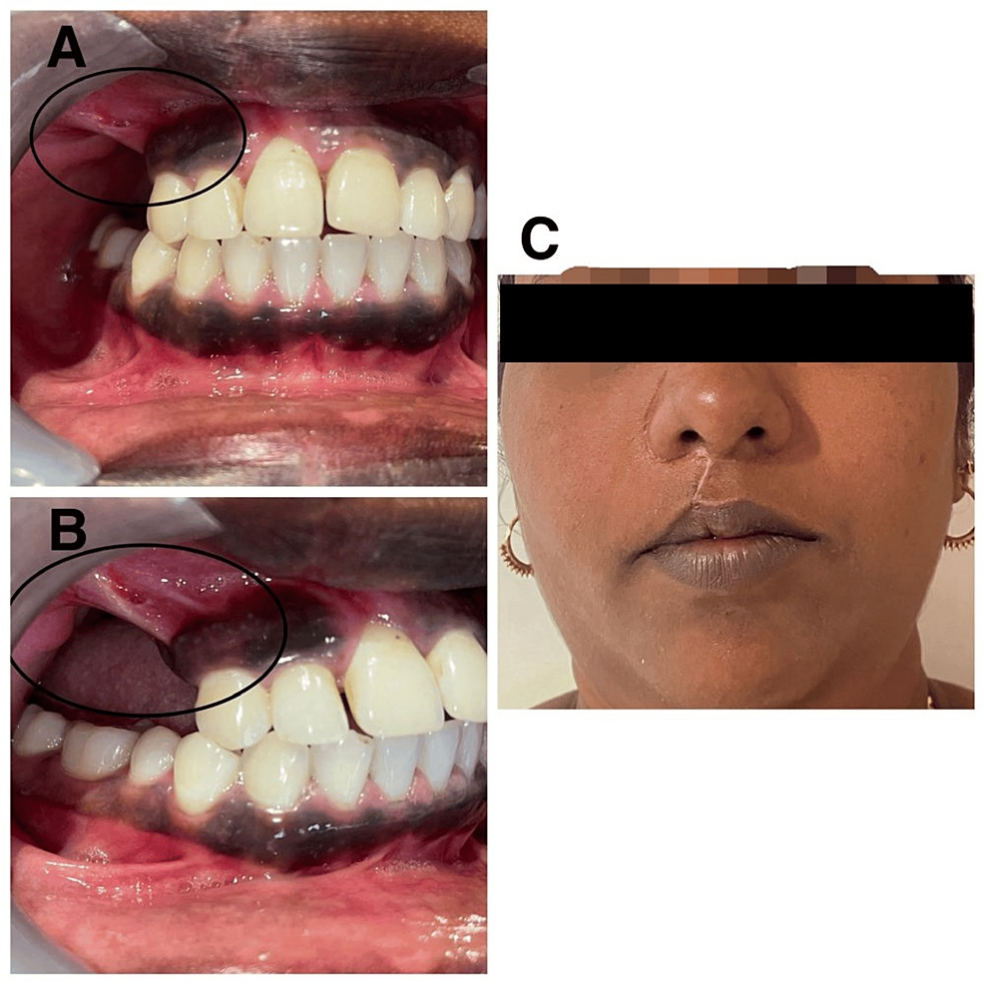
Postoperative photographs (A) Intraoral frontal. (B) Intraoral right side. (C) Extraoral.

**Figure 8:**
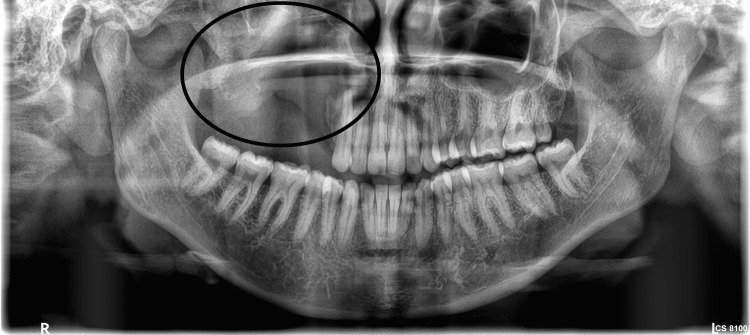
Postoperative radiograph

**Figure 9:**
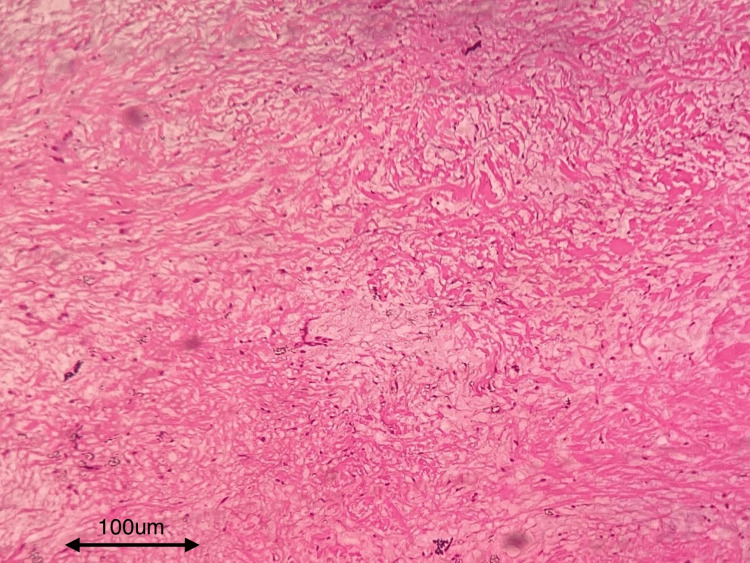
Histopathological slide showing loose myxoid tissue H&E stain with 40x magnification. Bony tissue was used.

The patient was planned for surgical enucleation under general anesthesia after being deemed fit for the procedure. Under general anesthesia, nasoendotracheal intubation was performed. An extraoral Weber-Fergusson incision with Lynch modification was made. Full-thickness mucoperiosteal flap elevation was performed, exposing the tumor margins. Enucleation of the lesion was conducted, accompanied by the extraction of teeth 14, 16, and 17. The margins were chemically cauterized using an electrocautery. Layer-wise closure was achieved using 3-0 vicryl and 4-0 proline sutures (Figures 5, 6). At the 13-month follow-up, no recurrence was observed (Figures 7, 8). An excisional biopsy confirmed odontogenic fibromyxoma, revealing the proliferation of stellate to spindle-shaped cells exhibiting ovoid, spindle, or angular nuclei within a loose myxoid-connective tissue stroma made of delicate collagen fibers and showing numerous engorged capillary-sized vessels (Figure 9).

## Discussion

Odontogenic fibromyoma is a benign neoplasm of intraosseous origin, characterized by its local aggressiveness, high recurrence rate, and nonmetastasizing nature. It is most commonly observed in the mandible, with a higher prevalence in females than males, typically occurring in the third and fifth decades of life. Thoma and Goldman suggested in 1947 that the degeneration of connective tissue tumors gives rise to myxoma [10]. However, myxoma of the facial bones is actually an odontogenic fibroma that underwent myxomatous degeneration. On the other hand, Zayet and Eiid propose that the tumor may develop as a result of mesenchymal resting growing inside the alveolar bone [11]. Fibromyxoma can occur at two different sites: (a) in facial bones and (b) in soft tissue myxomas in relation to the larynx, heart, parotid glands, and ear [12].

Radiologically, fibromyxoma can resemble various jaw lesions, potentially leading to misdiagnosis. The main entities to consider in the differential diagnosis are ameloblastoma, dentigerous cyst, odontogenic keratocyst (OKC), intraosseous hemangioma, aneurysmal bone cyst, and central giant cell granuloma. Ameloblastoma compartments typically exhibit rounded morphology, contrasting with the square or triangular areas seen in odontogenic fibromas. Additionally, ameloblastoma margins tend to be corticated, with no evidence of soft tissue invasion or interruption in the continuity of the cortex. Central giant cell granuloma is often located in the anterior mandible, presenting as a unilocular lesion with noncorticated margins [13]. Intraosseous hemangioma manifests as intraoral swelling and jaw numbness, displaying radiologically multilocular lesions with small loculations. Histologically, well-defined vascular spaces are evident. OKC presents with swelling and cortical plate expansion, appearing radiologically as unilocular or multilocular radiolucencies with curved or coarse septae. Histologically, it exhibits corrugated para or orthokeratinized luminal epithelium. Aneurysmal bone cysts are typically asymptomatic, featuring cortices perforation and common root resorption. Multiple sinusoidal blood-filled spaces are observed [14]. Distinguishing features include fine and straight septa forming a tennis racquet pattern, with septa perpendicular to the margins and scalloping between roots [15].

Histopathologically, myxofibroma (MF) comprises fibroblasts and myofibroblasts dispersed within loose myxomatous connective tissues, rich in acid mucopolysaccharides. While minimal pleomorphism may exist, it does not correlate with recurrence frequency. The balance of collagen and mucoid content determines whether it is termed MF or fibromyxoma. Spindle, hyaline, and stellate cells are expressed, with stellate cells positive for transferrin, S-100, vimentin, and alpha-1 AT [16]. Grossly, the myxomas are grayish-white to yellow in color, semi-solid, well-delineated, and semi-solid in consistency [17]. As fibromyxomas are typically resistant to radiation, surgical intervention, including curettage, enucleation, or radical resection, is the primary treatment. Recurrence, occurring within the first two years posttreatment, is common, with rates ranging between 25% and 40% [18].

In our case, we opted for conservative treatment due to the patient’s young age and the fact that she was breastfeeding. Additionally, lesions in the maxilla have a lower recurrence rate compared to mandibular lesions, as observed in a study conducted by Martins et al. [9,19]. The management of the lesion is influenced by multiple factors, including size, site, clinical behavior of the lesion, and the patient’s age. The conservative approach should be considered as it has less morbidity and improves the patient’s quality of life. Adjuvant procedures such as the application of Carnoy’s solution and peripheral osteotomy also decrease the incidence of recurrence and yield a more acceptable postoperative result. With almost 13 months of follow-up, no recurrence has been observed in our patient.

## Conclusions

Fibromyxomas of the jaws are not uncommon, although lesions in the maxilla are rare. Establishing a diagnosis can be challenging; therefore, radiographs and histopathological analyses are essential. Conservative treatment may be appropriate for young patients, with long-term follow-up recommended.
